# Core decompression combined with local DFO administration loaded on polylactic glycolic acid scaffolds for the treatment of osteonecrosis of the femoral head: a pilot study

**DOI:** 10.1186/s40360-023-00682-x

**Published:** 2023-09-05

**Authors:** Kaveh Gharanizadeh, Ali Mohammad Sharifi, Hamed Tayyebi, Razieh Heidari, Shayan Amiri, Sajad Noorigaravand

**Affiliations:** 1 Bone and Joint Reconstruction Research Center, Department of Orthopedics, School of Medicine, Iran University of Medical Sciences, Tehran, Iran; 2 Department of Pharmacology, School of Medicine, Iran University of Medical Sciences, Tehran, Iran; 3 Shohadaye Haftom-e-Tir Hospital, School of medicine, Iran University of Medical Sciences, Tehran, Iran; 4 Department of Radiology, Iran University of Medical Sciences, Tehran, Iran

**Keywords:** Osteonecrosis, Femoral head, Core decompression, Deferoxamine, PGLA

## Abstract

**Background:**

Deferoxamine (DFO) angiogenesis induction potential has been demonstrated in earlier studies, but not in the osteonecrosis of the femoral head (ONFH). In this study, we evaluated the outcome of ONFH treated with combined core decompression and local DFO administration loaded on Polylactic Glycolic Acid (PLGA).

**Patients and methods:**

In a pilot experimental study, six patients (10 hips) with early-stage non-traumatic ONFH were treated by core decompression, and concurrent injection of local DFO loaded on PLGA scaffold into the subchondral femoral head. Outcome measures were evaluated before the surgery and 12 and 24 months after the surgery and included visual analog scale (VAS) for pain, modified Merle d’Aubigné-Postel (MAP) score for hip function by MRI, and rate of osteonecrosis assessed by the modified.

**Results:**

The mean MPA score was 14.7 ± 1.16 before the surgery and 16.7 ± 1.41 one year after the surgery (P = 0.004). The mean VAS for pain was 4.7 ± 1.25 before the surgery and 1.8 ± 1.03 one year after the surgery (P = 0.005). The mean Kerboul angle was 219 ± 58.64 before the operation and 164.6 ± 41.82 one year after the operation (P < 0.001). Osteonecrosis progression or collapse was not seen in any of the patients at the final follow-up. No postoperative side effect attributed to the DFO was noticed, as well.

**Conclusion:**

In short-term follow-up, combined core decompression and local DFO administration not only prevent the progression of ONFH but also reduces the rate of osteonecrosis significantly. However, future controlled studies are required to confirm the present results.

**Trial registration:**

IRCT20161121031003N3, 16/04/2019.

## Background

In young individuals, osteonecrosis of the femoral head (ONFH) is the leading cause of hip arthroplasty due to its disabling nature, usually occurring in the most productive years of life. It is caused by insufficient blood supply of the femoral head and subsequent death of the osteocytes. Traumatic and nontraumatic etiologies have been introduced for ONFH. Corticosteroid consumption and chronic alcohol intake are the most common nontraumatic etiologies of ONFH. In about 30% of the patients, ONFH is idiopathic [1]. Early diagnosis and treatment are the keys to the successful preservation of the hip joint. If left untreated, it progresses to secondary hip arthritis, and arthroplasty remains the only therapeutic option [2]. Core decompression of the hip is the most widely used surgical procedure for the treatment of ONFH in the early stage, which induces new bone formation by decreasing the intraosseous pressure and increasing blood flow to the necrotic area of the femoral head [3, 4]. However, the result of core decompression is markedly dependent on the etiology of ONFH and characteristic features of the lesion, such as the size and location, so that an overall success rate of 40–80% has been reported in different studies at 2–7 year follow-up [5]. For this reason, many efforts are being made to optimize the outcome of core decompression. Insertion of bone grafts (vascularized or non-vascularized) to prevent collapse and supplementation with bone-marrow cells [6], demineralized bone matrix, or bone morphogenetic proteins to enhance bone repair are some of these efforts [7].

Angiogenesis factors such as vascular endothelial growth factor (VEGF), angiopoietin-1, fibroblast growth factor-2 have recently attracted attention in the treatment of ONFH [8]. Hypoxia-inducible factors (HIFs) are also acknowledged in the induction of angiogenesis. Therefore, factors that inhibit HIF degradation contain angiogenesis-inducing potential [9]. Deferoxamine (DFO) is an iron chelator that inhibits the degradation of HIF-1α, thereby improving the vascularization process [10]. A preliminary study in the excremental model showed that core decompression combined by the DFO administration was associated with more blood vessels and higher expression of antigenic factors in comparison with core decompression alone in the treatment of steroid-induced osteonecrosis of rabbit femoral heads [11].

Polylactic Glycolic Acid (PLGA) is one of the most successful FDA-approved biodegradable polymers used for controlled drug delivery systems. PLGA nanoparticles create porous structures that protect drugs from degradation and enhance their stability. For these characteristics, PLGA has been widely used as a drug carrier in recent studies [12].

In this pilot study, we aimed to evaluate the effect of core decompression combined with DFO loaded PLGA system in the treatment of patients with early stages ONFH.

## Patients & methods

This pilot experimental study was approved by the review board of our institute under the code of IR.IUMS.FMD.REC.1397.281. Patients provided written consent before participation in the study. The protocol of the study was registered on the Iranian Registry of Clinical Trials under the code IRCT20161121031003N3, 16/04/2019. The diagnosis of ONFH was made using conventional radiography and MRI. CT scan was used to rule out joint surface collapse. The inclusion criteria were the age of > 18 years and stage 2 lesions according to the ARCO classification (Association Research Circulation Osseous classification) [13]. Patients with traumatic ONFH and patients who were taking corticosteroids or immunosuppressive medications at the time of study, were excluded. Patients with underlying disorders adversely affecting the improvement of osteonecrosis, such as rheumatologic disease, collagen disorders, vascular diseases, chronic heart failure, renal failure, diabetes, etc., were excluded as well. Finally, six patients (10 hips) were included in this study.

The study population included three males and three females with the mean age of the 35 years (range 30–40). The lesion was bilateral in four patients and unilateral in two patients. The ONFH was corticosteroid-induced in four patients and idiopathic in two patients.

### Surgical procedure and postoperative protocol

**Fig. 1:**
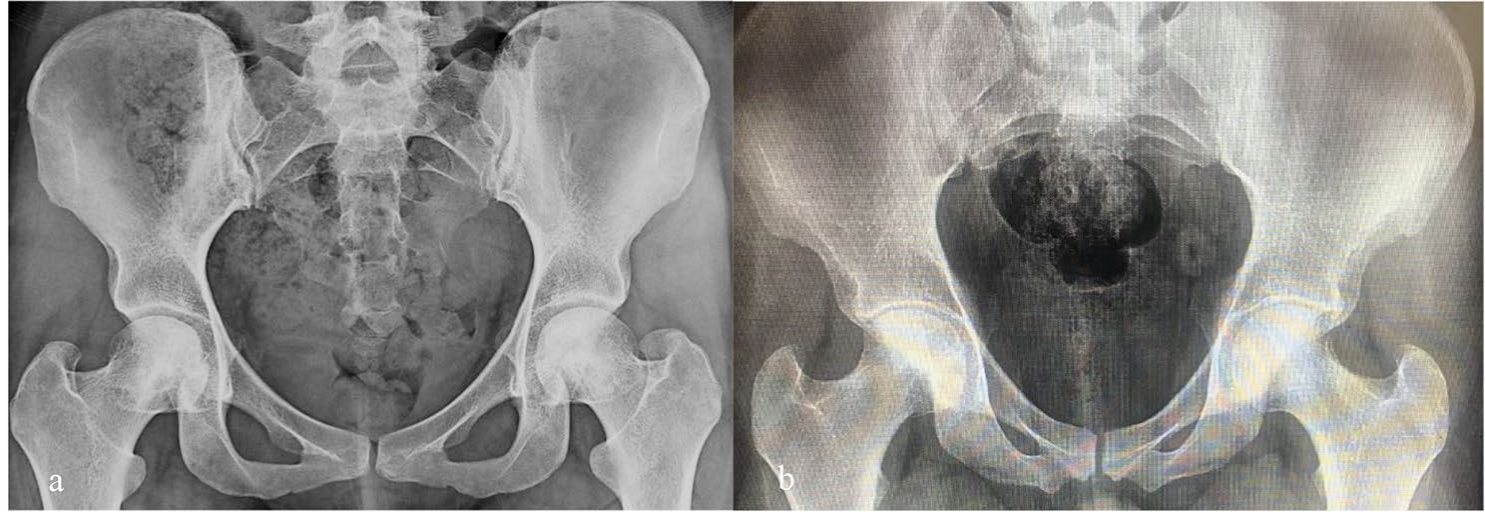
Preoperative (**a**) and (**b**) postoperative Radiography

**Fig. 2:**
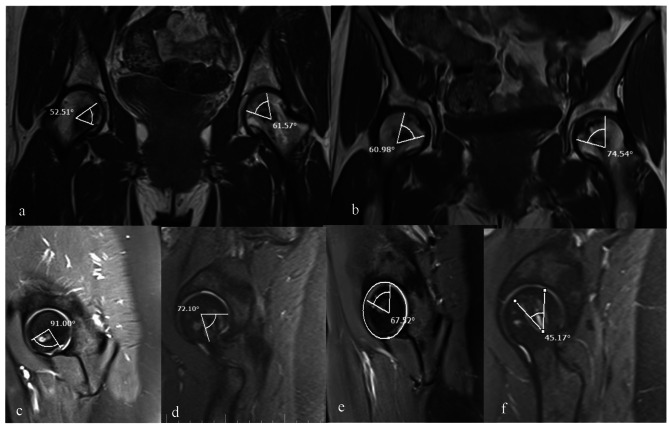
Preoperative (**a**) and (**b**) postoperative MRI, preoperative (**c**) and (**d**) postoperative MRI of right femoral head, preoperative (**e**) and (**f**) postoperative MRI of left femoral head

In the operating room and under spinal anesthesia, the patient was placed on the orthopedic table in the supine position. Under the fluoroscopic guidance, a minimal incision was made with respect to the site of osteonecrosis, which was determined on the preoperative radiographs (Fig. 1) and MRI (Fig. 2). Then, multiple drilling technique was implemented to create 3–4 tunnels in the osteonecrotic areas using a 4 mm cannulated drill. Immediately after the creation of tunnels, the PLGA-DFO system was injected into the subchondral portion of the femoral head using an 16G epidural needle. Pharmacologists created the PLGA-DFO vial in sterile conditions for surgical use. For this purpose, PLGA scaffolds with a molecular weight of 25 KDs prepared by salt-leaching technique (Sigma-Aldrich, USA) were loaded with 250 mg. The patients were discharged after 24 h. Up to six postoperative weeks, weight-bearing with a cane was allowed as much as tolerated. After that, full weight-bearing was allowed without using a cane. The patients were visited two weeks after the operation to check the wound and for radiographic evaluation of the lesion. Later follow-up visits were performed every two months.

### Outcome measures

In addition to evaluating the outcomes before the surgery, we conducted follow-up evaluations at 12 months and 24 months after the surgery Primary outcome measures were visual analog scale (VAS) for hip pain and modified Merle d’Aubigné-Postel (MAP) score for the hip function. VAS for pain ranged from 0 to 10, and a higher score indicated greater pain intensity. MAP score ranged between a minimum score of 3 and a maximum score of 18. A higher score was indicative of better function. Secondary outcome measures were the evaluation of the rate of osteonecrosis and incidence of collapse. The rate of osteonecrosis was assessed by the evaluation of the modified Kerboul angle, defined as the sum of angles formed by the length of the femoral head lesion and the center of the femoral head on the mid-coronal and mid-sagittal cuts of MRI [14]. The incidence of the collapse was assessed using CT scans.

### Statistical analysis

SPSS for Windows, version 16 (SPSS Inc., Chicago, Ill., USA) was used for statistical evaluations. Descriptive data were presented as mean ± standard deviation (SD) for quantitative variables and number (percentage) for qualitative variables. Normality distribution of data was checked with a Kolmogorov–Smirnov test. A paired t-test or its nonparametric counterpart (Wilcoxon signed-rank test) was used to compare paired data. P < 0.05 was considered significant.

## Results

The mean MPA score was 14.7 ± 1.16 before the surgery and 16.7 ± 1.41 one year after the surgery. This difference was statistically significant (P = 0.004). The mean VAS for pain was 4.7 ± 1.25 before the surgery and 1.8 ± 1.03 one year after the surgery. This difference was also statistically significant (P = 0.005). The mean Kerboul angle was 219 ± 58.64 before the operation and 164.6 ± 41.82 one year after the operation. This difference was statistically significant, as well (P < 0.001). The preoperative and postoperative outcome measures of the hips are demonstrated in Table 1. Comparison of the average outcome measures before and 12 and 24 months after the operation is summarized in Table 2.

**Table 1:** Preoperative and one-year postoperative outcome measures of the hips with ONFH treated with core decompression combined with DFO loaded PLGA system

ID	Preoperative MPA score	Final MPA score	Preoperative VAS	Final VAS	Preoperative Kerboul angle	Final Kerboul angle
**1**	14	17	6	2	261	216
**2**	16	18	4	1	301	200
**3**	15	17	6	2	240	190
**4**	15	17	6	2	210	170
**5**	15	17	5	3	284	206
**6**	12	13	6	4	243	169
**7**	15	18	3	1	207	155
**8**	15	17	4	1	145	122
**9**	16	17	3	1	119	83
**10**	14	16	4	1	180	135

ONFH: osteonecrosis of the femoral head; DFO: Deferoxamine; PLGA: Poly lactic-glycol acid; MPA: Merle d’Aubigné-Postel; VAS: visual analog scale. Data are presented as mean ± SD

**Table 2:** Comparison of preoperative and one-year postoperative outcome measures in ONFH treated with core decompression combined with DFO loaded PLGA system

Variable	Before the operation (n = 10 hips)	One year after the operation (n = 10 hips)	twe year after the operation (n = 10 hips)	P-value
**MPA score**	14.7 ± 1.16	16.7 ± 1.41	17.8 ± 1.44	0.004
**VAS for pain**	4.7 ± 1.25	1.8 ± 1.03	1.5 ± 1.01	0.005
**Kerboul angle (º)**	219 ± 58.64	164.6 ± 41.82	-	< 0.001

ONFH: osteonecrosis of the femoral head; DFO: Deferoxamine; PLGA: Poly lactic-glycol acid; MPA: Merle d’Aubigné-Postel; VAS: visual analog scale. Data are presented as mean ± SD

Osteonecrosis progression was not seen in any of the patients after the operation. The postoperative collapse was not seen in the postoperative radiograph (Fig. 1) and CT scan of any patients, as well. No postoperative complications such as wound healing problems and infection were recorded in any of the patients during the one-year follow-up period. No side effect attributed to the DFO was noticed, as well.

## Discussion

In this study, we evaluated the effect of core decompression combined with the injection of DFO loaded PLGA system on the treatment of early-stage ONFH. One year after the operation, the patients showed a significant reduction in the rate of necrosis. No collapse occurred during the follow-up period of the study (Fig. 1). The pain was significantly reduced in all patients. Also, the hip function was significantly improved in all patients. Two years after the operation, the rate of necrosis didn’t have any progress and also the pain of all patients was significantly reduced and no collapse seen too (Fig. 2). In addition, all patients experienced a significant improvement in hip function.

In earlier investigations, the usefulness of DFO for the treatment of the femoral head osteonecrosis model has been demonstrated in the experimental model. Li et al. examined whether administration of local DFO can promote angiogenesis and bone repair in rabbit models of early-stage steroid-induced ONFH. The rabbits were divided into three groups, including no treatment, core decompression, and core decompression in combination with local DFO administration. Six weeks after the operation, microvessel analysis showed more blood vessels in the DFO group compared to the other groups. The expression of angiogenesis-inducing genes such as HIF-1α and VEGF was also higher in the DFO group than in the other groups. In addition, the DFO group indicated a larger volume of new bone formation than the core decompression group. Accordingly, they concluded that DFO administration could be beneficial for the treatment of early-stage ONFH [15]. Sheng et al. examined the effect of combined Alendronate and DFO for preventing steroid-induced osteonecrosis of the femoral head and inducing bone regeneration in the rat model. Thirty-six rats were randomly assigned to the three study groups, including the combined alendronate and DFO group, alendronate only group, and the control (placebo) group. Eight weeks after the induction of osteonecrosis, Alendronate combined with the DFO group demonstrated higher expression of osteocalcin and VEGF and upregulated signal factors of HIF-1*α* and *β*-catenin, and decreased level of RANKL. In addition, bone volume, trabecular number and separation, and trabecular thickness were further improved in this group, while the ratio of osteocyte lacunae was lower. They concluded that combined Alendronate and DFO have positive effects on regulating bone resorption and regeneration, thereby preventing glucocorticoid-induced osteonecrosis of the femoral head [15]. DFO has also been used for inducing angiogenesis in other locations such as the mandible [16]. Localized DFO injections have also proved to remediate the associated severe vascular diminution caused by radiotherapy [17].

While core decompression is widely used for the treatment of human ONFH, its combination with local DFO administration has not been examined in any earlier studies. Although core decompression is acknowledged as the most successful treatment of early-stage ONFH [18], a considerable number of patients will convert to total hip arthroplasty (THA) after a minimum follow-up of two years [19]. According to the study of Etemadifar et al., 12 months after the operation, the stage of ONFH progressed in three out of 14 patients who were treated with core decompression (from II_A_ to II_B_), and the patients showed flattening of the femoral head [20]. In the present study, the follow-up period was not long enough to evaluate conversion to THA. However, the stage of ONFH was not progressed in any of the patients. This result could support the efficacy of local DFO administration in augmenting the outcome of core decompression in the treatment of ONFH. Further, studies have shown that in most cases, hips with Kerboul angles less than 250 degrees produce satisfactory results. Almost all hips with Kerboul angles above 250 degrees collapsed [21]. According to our results, the mean Kerboul angle was significantly decreased. Therefore, core decompression combined with local administration of DFO loaded into a polylactic glycolic acid scaffold for the treatment of osteonecrosis that we performed in this study, could have the perfect effect of reducing the need for THA. The present study had several limitations. The main limitation of the study is the absence of the control group. The second limitation of the study is the small number of patients. In addition, augmentation of angiogenesis was not confirmed by molecular examinations and microvessel analysis by immunohistochemical staining. In spite of the fact that this was the first study to use this method, it had no adverse effect. Therefore, future complementary controlled studies are required to confirm the effectiveness of core decompression combined with local DFO administration in the treatment of ONFH.

## Conclusion

The combination of core decompression and local DFO administration not only prevents ONFH progression, but also reduces osteonecrosis rates significantly. Further, it improves the functions of the patients as well as relieves their pain. Therefore, it could be regarded as an effective procedure for the treatment of early-stage ONFH. The present results need to be confirmed by further controlled studies.

## Data Availability

This study’s data support its findings, and these are available in the article.

## List of abbreviations

DFO: Deferoxamine
ONFH: Osteonecrosis of the femoral head
PLGA: Polylactic Glycolic Acid
VAS: Visual analog scale
MAP: Merle d’Aubigné-Postel
VEGF: Vascular endothelial growth factor
ARCOC: Association research circulation osseous classification
SD: Standard deviation
