# Correction: Core decompression combined with local DFO administration loaded on polylactic glycolic acid scaffolds for the treatment of osteonecrosis of the femoral head: a pilot study

**DOI:** 10.1186/s40360-023-00691-w

**Published:** 2023-10-17

**Authors:** Kaveh Gharanizadeh, Ali Mohammad Sharifi, Hamed Tayyebi, Razieh Heidari, Shayan Amiri, Sajad Noorigaravand

**Affiliations:** 1 Bone and Joint Reconstruction Research Center, Department of Orthopedics, School of Medicine, Iran University of Medical Sciences, Tehran, Iran; 2 Department of Pharmacology, School of Medicine, Iran University of Medical Sciences, Tehran, Iran; 3 Shohadaye Haftom-e-Tir Hospital, School of medicine, Iran University of Medical Sciences, Tehran, Iran; 4 Department of Radiology, Iran University of Medical Sciences, Tehran, Iran

**Correction: BMC Pharmacology and Toxicology (2023) 24:44**

10.1186/s40360-023-00682-x

Following publication of the original article [1], the authors identified an error in the author details. The correct author details are given below.

**The author details currently read**:

1. Bone and Joint Reconstruction Research Center, Department of Orthopedics, School of Medicine, Iran University of Medical Sciences, Tehran, Iran.
2. Department of Pharmacology, School of Medicine, Iran University of Medical Sciences, Tehran, Iran.
3. Shohadaye Haftom-e-Tir Hospital, School of medicine, Iran University of Medical Sciences, Tehran, Iran.
4. Tissue Engineering Group (TEG), National Orthopaedic Centre of Excellence in Research and Learning (NOCERAL), Department of Orthopaedic Surgery, Faculty of Medicine, Universiti Malaya, Kuala Lumpur, Malaysia.

**The author details should read**:

1. Bone and Joint Reconstruction Research Center, Department of Orthopedics, School of Medicine, Iran University of Medical Sciences, Tehran, Iran.
2. Department of Pharmacology, School of Medicine, Iran University of Medical Sciences, Tehran, Iran.
3. Shohadaye Haftom-e-Tir Hospital, School of medicine, Iran University of Medical Sciences, Tehran, Iran.
4. Department of Radiology, Iran University of Medical Sciences, Tehran, Iran.

The original article [1] has been corrected.
